# Identification of Renal Long Non-coding RNA RP11-2B6.2 as a Positive Regulator of Type I Interferon Signaling Pathway in Lupus Nephritis

**DOI:** 10.3389/fimmu.2019.00975

**Published:** 2019-05-03

**Authors:** Zhuojun Liao, Zhizhong Ye, Zhixin Xue, Lingling Wu, Ye Ouyang, Chao Yao, Chaojie Cui, Ning Xu, Jianyang Ma, Guojun Hou, Jiehua Wang, Yao Meng, Zhihua Yin, Ya Liu, Jie Qian, Chunyan Zhang, Huihua Ding, Qiang Guo, Bo Qu, Nan Shen

**Affiliations:** ^1^Shanghai Institute of Rheumatology, Renji Hospital, School of Medicine, Shanghai Jiao Tong University, Shanghai, China; ^2^Shenzhen Futian Hospital for Rheumatic Diseases, Shenzhen, China; ^3^State Key Laboratory of Oncogenes and Related Genes, Shanghai Cancer Institute, Renji Hospital, Shanghai, China; ^4^Collaborative Innovation Centre for Translational Medicine, Shanghai Jiao Tong University School of Medicine, Shanghai, China; ^5^Center for Autoimmune Genomics and Etiology (CAGE), Cincinnati Children's Hospital Medical Center, Cincinnati, OH, United States

**Keywords:** long non-coding RNA, type I interferon, lupus nephritis, RP11-2B6.2, SOCS1

## Abstract

**Objective:** Lupus nephritis (LN) is one of the most serious complications of systemic lupus erythematosus (SLE). Type I interferon (IFN-I) is associated with the pathogenesis of LN. Long non-coding RNAs (lncRNAs) have been implicated in the pathogenesis of SLE, however, the roles of lncRNAs in LN are still poorly understood. Here, we identified and investigated the function of LN-associated lncRNA RP11-2B6.2 in regulating IFN-I signaling pathway.

**Methods:** RNA sequencing was used to analyze the expression of lncRNAs in kidney biopsies from LN patients and controls. Antisense oligonucleotides and CRISPRi system or overexpression plasmids and CRISPRa system were used to perform loss or gain of function experiments. *In situ* hybridization, imaging flow cytometry, dual-luciferase reporter assay, and ATAC sequencing were used to study the functions of lncRNA RP11-2B6.2. RT-qPCR, ELISA, and western blotting were done to detect RNA and protein levels of specific genes.

**Results:** Elevated lncRNA RP11-2B6.2 was observed in kidney biopsies from LN patients and positively correlated with disease activity and IFN scores. Knockdown of lncRNA RP11-2B6.2 in renal cells inhibited the expression of IFN stimulated genes (ISGs), while overexpression of lncRNA RP11-2B6.2 enhanced ISG expression. Knockdown of LncRNA RP11-2B6.2 inhibited the phosphorylation of JAK1, TYK2, and STAT1 in IFN-I pathway, while promoted the chromatin accessibility and the transcription of SOCS1.

**Conclusion:** The expression of lncRNAs is abnormal in the kidney of LN. LncRNA RP11-2B6.2 is a novel positive regulator of IFN-I pathway through epigenetic inhibition of SOCS1, which provides a new therapeutic target to alleviate over-activated IFN-I signaling in LN.

## Introduction

Systemic lupus erythematosus (SLE) is a heterogeneous autoimmune disorder characterized by the occurrence of a wide range of autoantibodies and organ damage and predominantly affects women at childbearing age (1, 2). Lupus nephritis (LN) is considered one of the most prevalent and serious complications of SLE with high morbidity and mortality (3–5). Routine therapies for LN are largely based on steroids and non-specific immunosuppressants, most of which are prone to irreversible gastric-ulcer and life-threatening leucopenia (6). In-depth investigation of the molecular mechanisms for the dysregulation of immune responses will facilitate the discovery of new therapeutic targets with reduced adverse effects and improved curative efficacy.

Long non-coding RNAs (lncRNAs) (>200 nucleotides in length) are a class of widespread transcriptional outputs (7, 8), and have been recognized as important regulators in many physiological or pathological processes. Our previous studies have indicated a link between the dysregulation of lncRNAs in peripheral blood mononuclear cells (PBMCs) and disease activity of SLE. For example, linc0949 is decreased in PBMCs of SLE patients and the expression of linc0949 correlates with SLEDAI-2K scores and is associated with SLE-related organ damage such as LN (9). What's more, in another study, we found that NEAT1 was an early response gene downstream of Toll-like receptor 4 (TLR4) and could regulate the activation of MAPK pathway in monocytes of SLE patients (10). So far, many studies considering the functions of lncRNAs in the pathogenesis of SLE have been done mainly in immune cells. The expression profile and the functions of lncRNAs in kidney tissues of LN are still not clear.

Among numerous pathogenic signaling pathways recognized in LN, over-activation of type I interferon (IFN-I) responses is associated with disease progression and prognosis (11, 12). While, IFNAR deficiency protects mice from suffering severe lupus nephritis (13, 14). Many molecules that have the activity of blocking IFN-I signaling pathway have been developed to ameliorate the symptoms of SLE, such as JAK inhibitor (tofacitinib) (15), monoclonal antibodies targeting IFN alpha (sifalimumab) (16) and IFN-I receptor (anifrolumab) (17).

Thus, in this paper, we examined the expression profile of lncRNAs in the kidney biopsies of LN patients. Among all the differentially expressed lncRNAs, we selected lncRNA RP11-2B6.2 for further studies. Our results suggest that lncRNA RP11-2B6.2 functions as a positive regulator in IFN-I signaling pathway through epigenetic inhibition of SOCS1 gene. Our findings provide new insights into the function of abnormally expressed renal lncRNAs in LN and a potential therapeutic target used to interfere overactivated IFN-I signaling pathway.

## Materials and Methods

### Study Subjects

For RNA sequencing, twenty-two LN kidney biopsies and seven control samples were collected at Renji Hospital, Shanghai Jiao Tong University School of Medicine after the patients signed the informed consent form. All LN patients fulfilled the American College of Rheumatology (ACR) criteria for SLE in 2009 (18) and for LN in 2012 (4). All control samples were from the para-carcinoma tissues of kidney tumor patients with no history of autoimmune diseases or treatment with immunosuppressive agents. SLE Disease Activity Index (SLEDAI) (19) and LN Activity (A) / Chronicity (C) Index (20) were assessed for each LN patient at the time of renal biopsy. Characteristics of LN patients were listed in Table 1. Additional LN patients (*n* = 30) and age and sex matched healthy volunteers were recruited for the quantification of lncRNA RP11-2B6.2 in PBMCs by qPCR. All procedures and protocols were reviewed and approved by the Research and Ethics Board of Renji Hospital.

**Table 1:** Demographic, clinical, and laboratory characteristics of LN patients.

**Characteristics**	Acute renal lesion^*^ (*n* = 13)	Chronic renal lesion^*^ (*n* = 9)
Sex, male/female (number)	2/11	0/9
Age, mean ± SD (years)	39.62 ± 9.26	41.11 ± 7.69
Disease duration, mean ± SD (months)	42.62 ± 32.90	33.78 ± 29.24
SLEDAI score^*^, mean ± SD	12.77 ± 6.88	14.44 ± 3.09
Anti-dsDNA, no. positive/negative	10/3	5/4
Low complementary, no. positive/negative	4/9	3/6
Proteinuria, no. positive/negative	11/2	7/2
Steroids, no. taken ^†^	6	3
≤ 15 mg/day	4	4
15–40 mg/day(≈0.5 mg/kg/d)	3	2
40–120 mg/day(≈1–2 mg/kg/d) >120 mg/day	0	0
Disease-modifying anti-rheumatic drugs (DMARDs) no. taken. Yes/No ^†^	8/5	5/4

* *NIH Lupus Nephritis Activity (A) and Chronicity (C) Score System: A 0-24, C 0-12. SLE Disease Activity Index (SLEDAI): 0–4 inactive, 5–9 mild active, 10–14 moderate active, >14 severe active*.

† *Prednisone or other steroids converted to prednisone equivalents (e.g., 5 mg of prednisone was considered equivalent to 4mg of methylprednisolone, 0.75 mg of dexamethasone or 20 mg of hydrocortisone). DMARDs received by LN patients included: cyclophosphamide (CTX), methotrexate (MTX), azathioprine (AZA), tacrolimus (FK506), cyclosporine A (CsA) and mycophenolate mofetil (MMF)*.

### RNA Sequencing

Kidney biopsies or cell pellets were homogenized and stored in TRIzol Reagent (life technologies) at −80°C until further processing. RNA was isolated according to manufacturer's instruction. Using Next Ultra RNA Library Prep Kit (NEB), cDNA libraries were prepared from isolated RNAs with end repair and adapter ligation. The resulting libraries were quantified with qPCR for Hiseq 4000 Sequencing Platforms (Illumina) and then pooled.

Raw reads were mapped to the human reference genome (UCSC genome browser hg19 assembly) using HISAT2 (v2.0.4). Transcript levels were computed using kallisto (v0.43.0) with reference gene annotations (Geocode version 19). Statistical normalization and visualization were performed in R (v3.3.2) using DESeq2 package (v1.14.1).

### Isolation of Peripheral Blood Mononuclear Cells

Peripheral blood samples (10ml) were obtained from study subjects. The samples were collected using tubes containing ethylene-diamine-tetraacetic-acid (EDTA) (Becton, Dickinson and Company). PBMCs were isolated using Ficoll-Paque PLUS by density gradient centrifugation as instructed by the manuscript (GE Healthcare Life Science).

### Plasmid Construction

To create an overexpression plasmid for lncRNA RP11-2B6.2, full-length lncRNA RP11-2B6.2 was amplified from human cDNA (PCR primers: forward 5′-CTTTTTATTTAGATGATATTAAAACTCAGAAGAATT-3′, reverse 5′-TTCTGATCATGAATTTTATCATCATTAAAAAC-3′) using KOD polymerase (TOYOBO), and inserted into pcDNA3.1(+)-5′-HA vector (Invitrogen) between XhoI and NotI (Thermo FD) using T4 ligase (Promega).

To create the luciferase reporter plasmid for SOCS1 promoter, upstream sequences (2kb) of SOCS1 transcriptional start site (TSS) were amplified from human genomic DNA (PCR primers: forward 5′-GCAATCCACTACGACTGGCT-3′, reverse 5′-CCCCTGCGCCAGTCTTTTAA-3′) and inserted into firefly PGL3-basic vector (Promega) between KpnI and MluI using T4 ligase (Promega).

### Cell Culture and Transfection

HeLa cells and HK2 cells were purchased from Cell bank of Shanghai Institutes for Biological Sciences, human renal mesangial cells (HRMCs) were purchased from ScienCell. All the cells were authenticated to be without contamination of mycoplasma. Hela cells were grown in DMEM (Gibco) + 10% FBS (Gibco), HMRCs were grown in Mesangial Cell Medium (MCM, ScienCell) + 10% FBS (Gibco), and HK2 cells were grown in advanced DMEM/F-12 (Gibco) +10% FBS (Gibco). All the cells were maintained at 37°C in a humidified incubator under an atmosphere of 5% CO_2_.

A day before transfection, cells were seeded in 24-well-plates at a density of 1 × 10^5^ cells/well in a total volume of 500 μl/well of complete medium. Transfection mixture (for one well) was prepared by combining 200nM of antisense oligonucleotides (ASOs) against lncRNA RP11-2B6.2 (#1: 5′-TTTGCCCATCGGAGGAAAGC-3′; #2: 5′-TAAGTCACCTGGGTTGGGCC-3′)/non-targeting scramble oligonucleotides (5′-TCTACTCGTCGCTACGTACC-3′) for loss of function experiments, or 200 ng of lncRNA RP11-2B6.2 overexpression/empty vectors for gain of function experiments, with transfection reagent (1 μl Lipofectamine RNAimax for loss of function experiments or 0.5 μl Lipofectamine 2000 for gain of function experiments, Invitrogen) in 200 μl serum free Opti-MEM medium (Gibco). The mixture was incubated at room temperature for 20 min before added into the cultures. Twelve to twenty-four hours post-transfection, cells were stimulated with IFN-I (PBL InterferonSource #11200, Universal Type I Interferon) at a concentration of 1000 U/ml for 6 h.

### Real-Time Quantitative PCR (RT-qPCR)

Total RNA was extracted using TRIzol Reagent. The concentration and quality of RNA were assessed by absorbance spectrometry (260/280nm) using Nanodrop-2000 (Thermo). Primers were listed in Supplementary Table 1. cDNA was prepared with PrimeScript RT reagent kit using the oligo-dT protocol (Takara), and qPCR was done using SYBR Premix Ex Taq (Takara) on a vii7 real time PCR instrument (Applied Biosystems). All experiments were performed in biological triplicates. Each transcript level was normalized by the housekeeping gene glyceraldehyde-phosphate dehydrogenase (GAPDH).

### Western Blotting

HeLa cells were transfected with antisense or scramble oligonucleotides as described previously and treated with IFN-I for 0min, 15min, 30min, and 1h before lysis with RIPA lysis buffer containing 1% halt protease and phosphatase inhibitor cocktail (Thermo). Protein concentration was determined with the Pierce BCA protein assay. Proteins were incubated at 95°C for 10 min under reducing condition. Then, 30 μg of protein lysates were loaded and separated by sodium dodecyl sulfate–polyacrylamide gel electrophoresis and transferred onto a PVDF membrane (Millipore). Blots were blocked with PBST buffer containing 5% bovin serum albumin (BSA; BBI Life Sciences), probed with different primary or secondary antibodies, and imaged with Super Signal West Pico Kit (Thermo). Antibodies used were listed in Supplementary Table 2. Protein expression levels were normalized by the housekeeping gene beta (β)-actin.

### Enzyme-Linked Immunosorbent Assay (ELISA)

To measure the secretion of CXCL10 proteins, the culture supernatants of HRMCs and HK2 cells were harvested at 12 h after stimulation with IFN-I, and measured by the human CXCL10 ELISA kit (Biolegend) according to the manufacturer's instruction. Briefly, samples were incubated at room temperature for 1 h, then incubated with detection antibody for 30min, signal was then developed by adding substrate solution. Absorbance was read at 450nm in an opaque 96-well plate reader after addition of the stopping solution.

### Dual-Luciferase Reporter Assay

One hundred nanogram of reporter constructs containing SOCS1 promoter sequences or interferon-stimulated response element (ISRE; Clontech) upstream of the firefly luciferase open reading frame were co-transfected with 8 ng of Renilla luciferase vector (Promega) and 200 nM of ASOs or scramble oligonucleotides into HeLa cells using Lipofectamine 2000. Before lysis, the transfected cells were treated with IFN-I as indicated in the figures. Normalized luciferase activity (ratio of firefly to Renilla) for each well was determined using the Dual-Luciferase Reporter Assay System (Promega), measured by CENTRO XS3 LB 960 microplate luminometer (Berthold Technologies).

### RNA Scope

Locus-specific RNA *in situ* hybridization was done using probes for the gene of lncRNA RP11-2B6.2 (568815589:94176658-94177657), and for HS-PP1B-positive/DapB-negative control (Advanced Cell Diagnostic) according to manufacturer's instruction. HeLa cells were fixed with 4% paraformaldehyde (PFA; GoogleBio) and stored in 100% ethyl alcohol (Sino Pharm). Renal biopsies obtained from 3 LN patients were incubated in fixative solution (ACD). After hybridization with preheated probe and staining with hematoxylin, the slides were analyzed using an optical microscope (Leica DM500).

### CRISPR-dCas9-Mediated Inhibition/Activation

A plasmid containing sequence encoding dCas9 protein fused with tag-cherry and KRAB domains (from Stanley Qi's lab, Stanford University) or a plasmid containing dCas9 protein fused with tag-GFP and VP64 domains (Addgene No. 61422) were used to perform loss (CRISPRi) or gain (CRISPRa) of function experiments. Appropriate sgRNAs were cloned into BsmBI (NEB) sites of the lenti-guide-puro plasmid (Addgene No. 52963) or guide-synergistic activation mediator (SAM) plasmid (from Zhang F lab, Massachusetts Institute of Technology). Individual sgRNAs with highly predicted specificity and efficiency were chosen from 2 genomic regions upstream of the TSS of lncRNA RP11-2B6.2: −50 to +1000 bp for CRISPRi (#1 AACATCAAACTTCCTTGGAT, #2 GGCCTTGTAATCAACAAGCA); −500 to −50 bp for CRISPRa: (#3 TCTGTAGCAAGTACACTGGA, #4 TAGAAAAGTCTGCATCCAGG). The plasmids were used at a concentration of 500 ng per well (sgRNA: dCas9 1:1) and packed into liposomes and transfected into HeLa cells for 48h (21).

### ImageStream Flow Cytometry

HeLa Cells with and without intervention were disposed into fixation/permeabilization buffer (eBioscience) and incubated in the presence of live/dead probe and primary antibodies: DAPI (Thermo, 1:1,000 dilution), STAT1-PE (Cell Signaling Technology, 1:50 dilution), pSTAT1-Alexa Fluor 647 (Cell Signaling Technology, 1:50 dilution). In total, up to 10,000 events were collected for each sample on ImageStream cytometer (Amnis). The collected images were analyzed with ImageStream data exploration and analysis software (Amnis). Nuclear translocation was quantitatively measured using similarity analysis on in-focus single cells, and expression levels were analyzed as the median fluorescence intensity adjusted by isotype IgG control (BD) (22).

### Assay for Transposase-Accessible Chromatin With High-Throughput Sequencing (ATAC-Seq)

ATAC-seq was performed using ATAC reagent kit (Buenrostro) following the manufacture's instruction. Briefly, Hela cells were resuspended in lysis buffer and in transposase reaction mixture successively. Purified DNA was subsequently amplified with a pre-determined optimal cycle number. All data were finally pooled and clustered for further analysis (23).

### Calculation of IFN Scores

The mean expression level of each representative ISG (LY6E, IFI27, and MX1) in the controls was subtracted by its expression level in each patient, and the remaining value was divided by the SD value for the ISG in controls to obtain the standardized expression level of the ISG gene. The standardized values of the three genes were then summed to obtain the IFN score for each patient to evaluate their overall activation of the IFN-I signaling pathway (24, 25). The mean IFN score of patients and controls was 14.098 (range −2.791–98.563) and 0.000 (range −2.667–5.472), respectively.

### Statistical Analysis

Experimental data were analyzed using Graphpad 5 software (version 5.01). Non-parametric Mann-Whitney U test was used to compare the differences between two groups, such as patients and controls. Two-tailed unpaired *t*-test was used to compare the gene expression levels and luciferase reporter activities between two groups. One-way ANOVA was used to analyze the differences among three groups. Spearman's test was used for correlation analysis. *P* < 0.05 was considered statistically significant.

## Results

### The Expression of LncRNA RP11-2B6.2 Is Increased and Correlated With ISGs in Kidney Tissues of LN Patients

**Figure 1:**
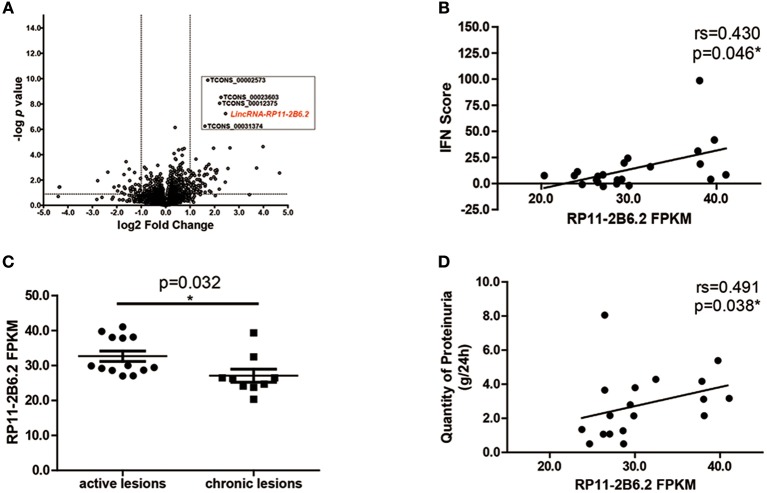
LncRNA RP11-2B6.2 Expression Is Increased in LN Patients. **(A)** 78 lncRNAs showed differential expression in transcriptome profiling of renal tissues from 22 LN patients compared to 7 controls. The vertical lines correspond to a 2-fold upregulation or downregulation, and the horizontal line represents *P* = 0.05. **(B)** Positive correlation between lncRNA RP11-2B6.2 levels and IFN scores in kidney biopsies of LN patients. **(C)** Patients were classified using the LN Activity/Chronicity Index, and the expression of lncRNA RP11-2B6.2 in LN patients with different disease activity was presented. **(D)** Positive correlation between lncRNA RP11-2B6.2 levels and concurrent quantity of 24-h proteinuria from LN patients. The figure **(B–D)** showed the number of fragments per kilobase of exon per million fragments mapped (FPKM). Non-parametric Mann–Whitney *U*-test was used in **(C)**. Spearman's test was used in **(B,D)**. ^*^P < 0.05.

To study the differential expression of lncRNAs in the kidney tissues of LN patients, we performed next-generation RNA sequencing using renal biopsy samples from 22 patients and 7 controls. As expected, a panel of universal ISGs were found increased in the samples of LN patients (Supplementary Figure 1), which indicates an over-activated IFN response in the kidney of LN. As to lncRNAs, 78 lncRNAs increased more than 2-fold in the samples from LN patients compared with those from controls. Five lncRNAs were selected for further analysis by their *p*-values (top 5 lowest) (Figure 1A). To explore the relationship between the expression levels of the 5 selected lncRNAs and the activation of IFN signaling pathway, we performed spearman's test using the expression data of the 5 lncRNAs and IFN scores calculated using the expression of ISGs. Among the 5 lncRNAs, lncRNA RP11-2B6.2 was the only one that was positively correlated with IFN scores (*r* = 0.430, *p* = 0.046, Figure 1B and Supplementary Table 3).

Next, we examined whether there was any relationship between the expression levels of lncRNA RP11-2B6.2 and clinical features in our cohorts. We found that lncRNA RP11-2B6.2 was expressed significantly higher in LN patients with active lesions than those in LN patients with chronic lesions (*P* < 0.05, Figure 1C). In addition, a positive correlation between the levels of lncRNA RP11-2B6.2 and the quantities of proteinuria was observed in patients presented concurrent abnormal levels of baseline 24-h urine protein (>0.5 g/d, *r* = 0.491, *P* = 0.038, Figure 1D). When considering the medication status, no differences were seen in the expression levels of lncRNA RP11-2B6.2 among the patients receiving various doses of glucocorticoids, or between the patients taking glucocorticoid plus secondary anti-rheumatic agents and the patients taking only glucocorticoid (Supplementary Figures 2A,B). In addition, we measured the expression levels of lncRNA RP11-2B6.2 in PBMCs of lupus nephritis patients using additional samples. We also found that lncRNA RP11-2B6.2 was expressed higher in PBMCs of lupus nephritis patients than in healthy controls (Supplementary Figure 3A). However, the difference of the expression levels of lncRNA RP11-2B6.2 in PBMCs between lupus nephritis patients and healthy controls was smaller than that in kidney tissues (Supplementary Figures 3A,B).

Collectively, the above results indicate that lncRNA RP11-2B6.2 is overexpressed and positively correlated with the expression of ISGs in the kidney tissues of LN patients.

### LncRNA RP11-2B6.2 Positively Regulates the Activation of IFN-I Signaling Pathway

We first studied if lncRNA RP11-2B6.2 could be induced by IFN-I. We stimulated HeLa cells with IFN-I and found that lncRNA RP11-2B6.2 was dramatically induced and peaked at 1h (Supplementary Figure 4A). The induction was also observed in human renal glomerular mesangial cells and tubular cell lines (HK2 cells, proximal tubular epithelial cells) (Supplementary Figures 4B,C and 5) (3).

**Figure 2:**
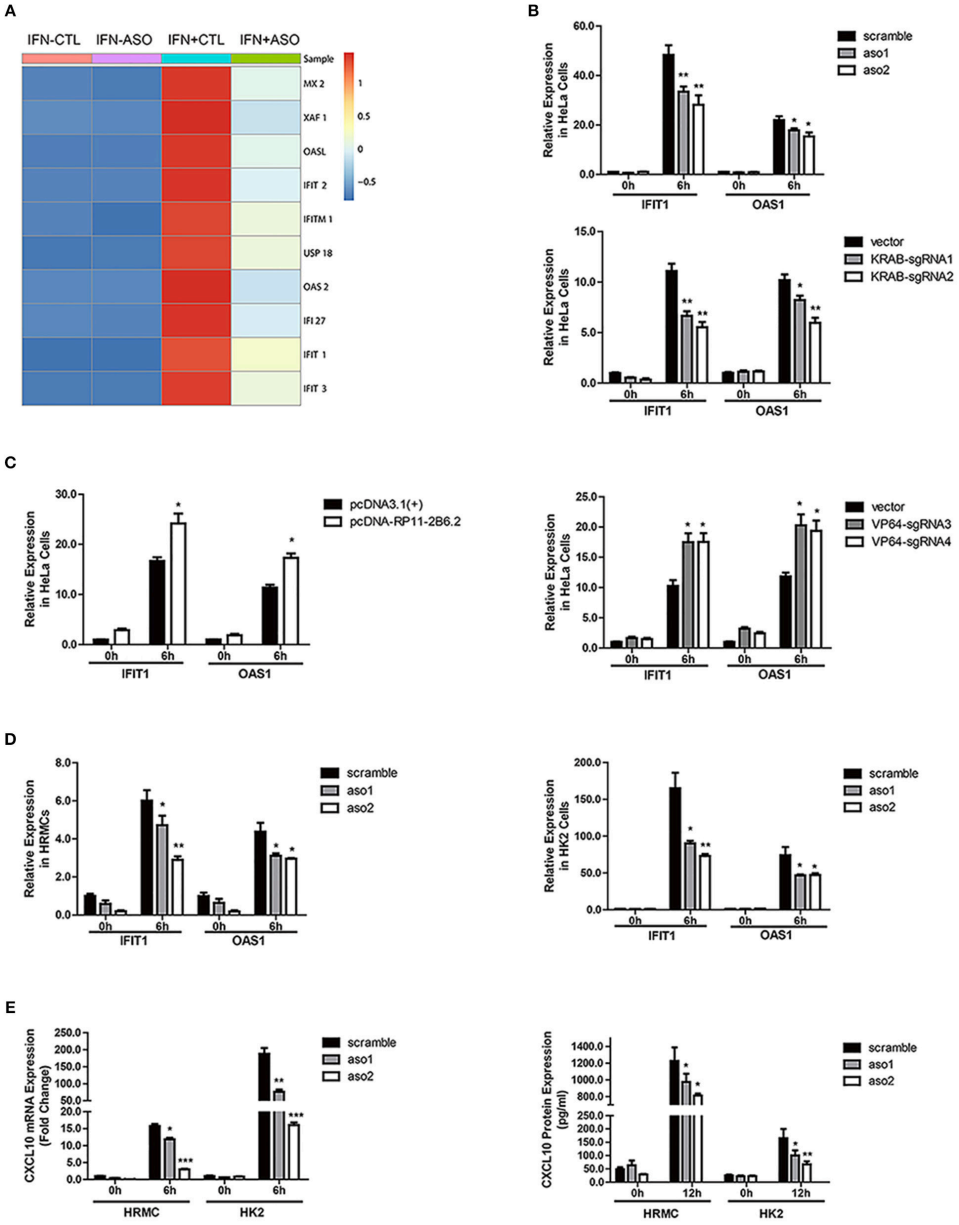
LncRNA RP11-2B6.2 Positively Regulates the Activation of IFN-I Pathway. **(A)** Expression changes of IFN-I-inducible genes in HeLa cells after down-regulation of lncRNA RP11-2B6.2 by ASOs. **(B)** HeLa cells were transfected with lncRNA RP11-2B6.2 ASOs (200 nM) or scramble (200 nM) for 12 h, and dcas9-KRAB (250 ng) together with sgRNA vectors (250 ng) for 48 h, then IFN-I was added. The cells were harvested after 6 h of stimulation. The relative expression of IFIT1 and OAS1 was detected with qPCR. **(C)** HeLa cells were transfected with lncRNA RP11-2B6.2 overexpression (200 ng) or empty pcDNA 3.1(+)-5′-HA vectors (200 ng) for 24 h, and dcas9-VP64 (250 ng) together with sgRNAs (250 ng) for 48 h, then IFN-I was added. The cells were harvested after 6 h of stimulation. The relative expression of IFIT1 and OAS1 was detected with qPCR. HRMCs and HK2 cells were transfected with lncRNA RP11-2B6.2 ASOs or scramble before IFN-I treatment. IFIT1 and OAS1 mRNA levels detected by qPCR after 6 h were shown in **(D)**. CXCL10 mRNA levels were detected by qPCR after 6 h of stimulation, and CXCL10 protein levels in the supernatants were detected by ELISA after 12 h of stimulation, data are shown in **(E)**. The group with the negative control or empty vector and without the addition of IFN-I, was set to “1.” ^*^P < 0.05, ^**^P < 0.01, ^***^P < 0.001.

To explore if lncRNA RP11-2B6.2 was involved in regulation of IFN-I signaling pathway, we analyzed the gene expression profile of IFN-I stimulated HeLa cells with or without silenced lncRNA RP11-2B6.2. Among 179 lncRNA RP11-2B6.2-regulated protein coding genes (more than 1.5-fold change and *p*-value < 0.05), downstream effector genes of IFN-I pathway (i.e., MX2, OASL, IFI27, IFIT1, IFIT3) were significantly overrepresented by GO functional pathway enrichment analysis (https://string-db.org/) (Figure 2A and Supplementary Figure 6). Then, we chose to measure IFIT1 and OAS1, two representative SLE-related ISGs (26) in HeLa cells, to verify the findings by RNA-seq. We used two different methods (antisense oligonucleotides and CRISPRi/dcas9-KRAB vector system) to downregulate the expression of lncRNA RP11-2B6.2 in Hela cells (Supplementary Figure 7). And we found that knockdown of lncRNA RP11-2B6.2 inhibited IFN stimulated expression of IFIT1 and OAS1 in Hela cells (Figure 2B). We also used two different methods (overexpression vector and CRISPRa/dcas9-VP64 vector system) to perform gain of function experiments (Supplementary Figure 6). We found that the upregulation of lncRNA RP11-2B6.2 increased the expression of IFIT1 and OAS1 in Hela cells stimulated with IFN-I (Figure 2C). Additionally, knockdown of lncRNA RP11-2B6.2 suppressed the expression of the two above-mentioned ISGs in HRMCs and HK2 cells (Figure 2D). As HRMCs and HK2 cells can release LN-related inflammatory chemokines CXCL10 (3), we subsequently revealed that IFN stimulated expression of CXCL10 was also reduced at both the mRNA and protein levels when lncRNA RP11-2B6.2 was downregulated in HRMCs and HK2 cells (Figure 2E).

**Figure 3:**
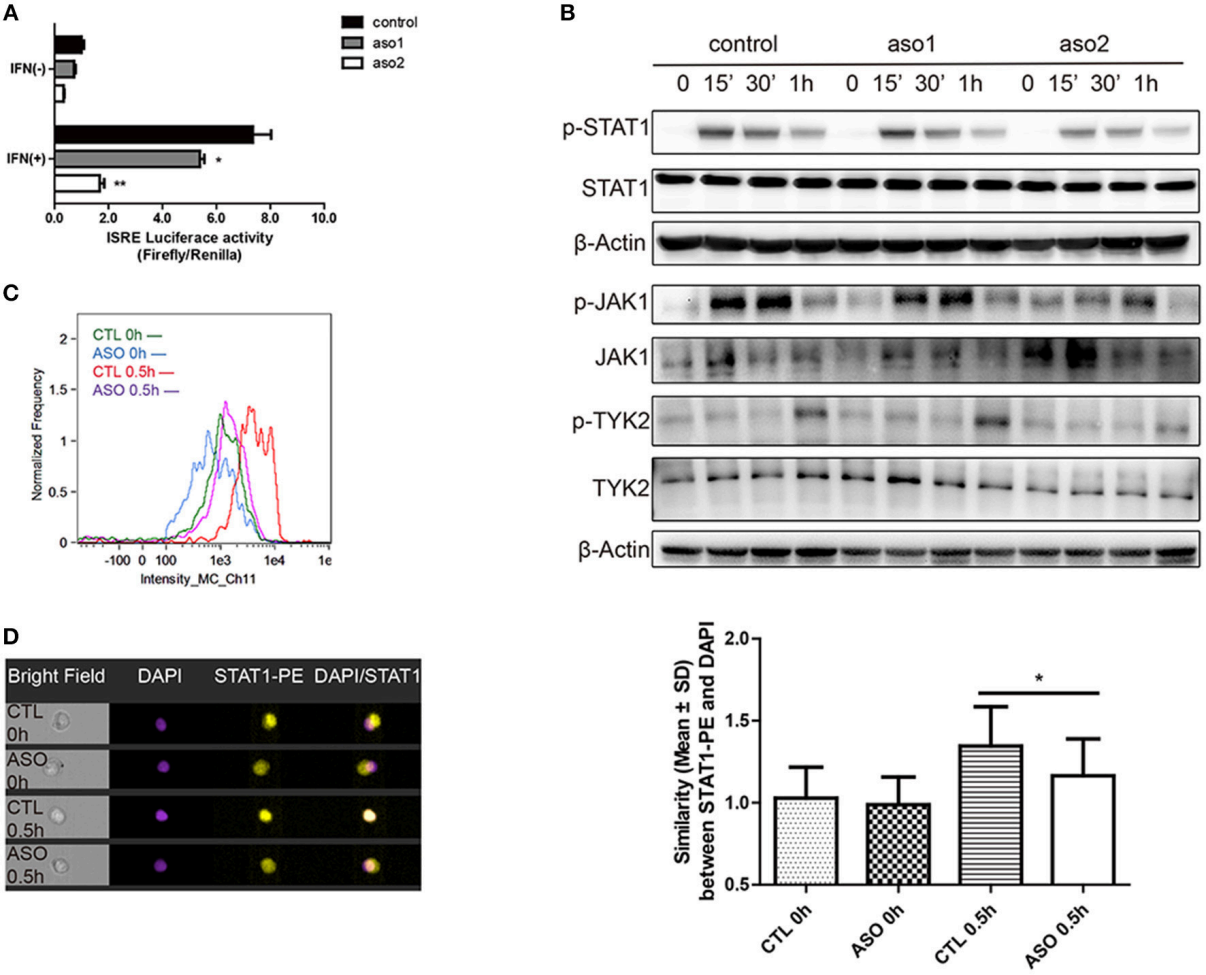
LncRNA RP11-2B6.2 Affects the Phosphorylation of Signaling Proteins in IFN-I Pathway. HeLa cells were transfected with lncRNA RP11-2B6.2-ASOs or scramble (200nM) for 12h. **(A)** Luciferase activity (Firefly/Renilla) of ISRE were analyzed after co-transfection with a reporter gene vector (100 ng). **(B)** Phosphorylation and total protein levels of TYK2, JAK1, and STAT1 were analyzed after stimulation of IFN-I for 0, 15min, 30min, and 1h. **(C)** Fluorescence intensity of p-STAT1 were analyzed after staining with AF647-conjugated antibody. **(D)** Similarity values between STAT1-PE and nucleus DAPI were analyzed after fixation and permeation. Values were means ± SEM from three independent experiments and *P*-values were analyzed with two-tailed unpaired *t*-test. ^*^P < 0.05, ^**^P < 0.01.

The activation of IFN-I signaling pathway includes the phosphorylation of TYK2, JAK1 and STAT proteins and the subsequent formation of a transcriptional factor complex ISGF3. ISGF3 finally transports into the nucleus and binds to the ISRE elements in the promoters of ISGs to initiate transcription (27). We evaluated the effect of lncRNA RP11-2B6.2 on the activation of IFN-I downstream signaling events by performing ISRE reporter gene assay. As expected, transiently silencing of lncRNA RP11-2B6.2 in HeLa cells substantially inhibited the IFN induced ISRE activity (Figure 3A). Consistently, downregulation of lncRNA RP11-2B6.2 by ASOs reduced the phosphorylation of STAT1, JAK1, and TYK2 (Figure 3B). We verified the results by imaging flow cytometry, since we observed a reduction of IFN-I-triggered STAT1 phosphorylation and nuclear translocation in lncRNA RP11-2B6.2-silenced HeLa cells (Figures 3C,D).

Collectively, these results demonstrate that lncRNA RP11-2B6.2 positively regulates the activation of IFN-I signaling pathway.

### LncRNA RP11-2B6.2 Suppresses the Transcription of SOCS1

**Figure 4:**
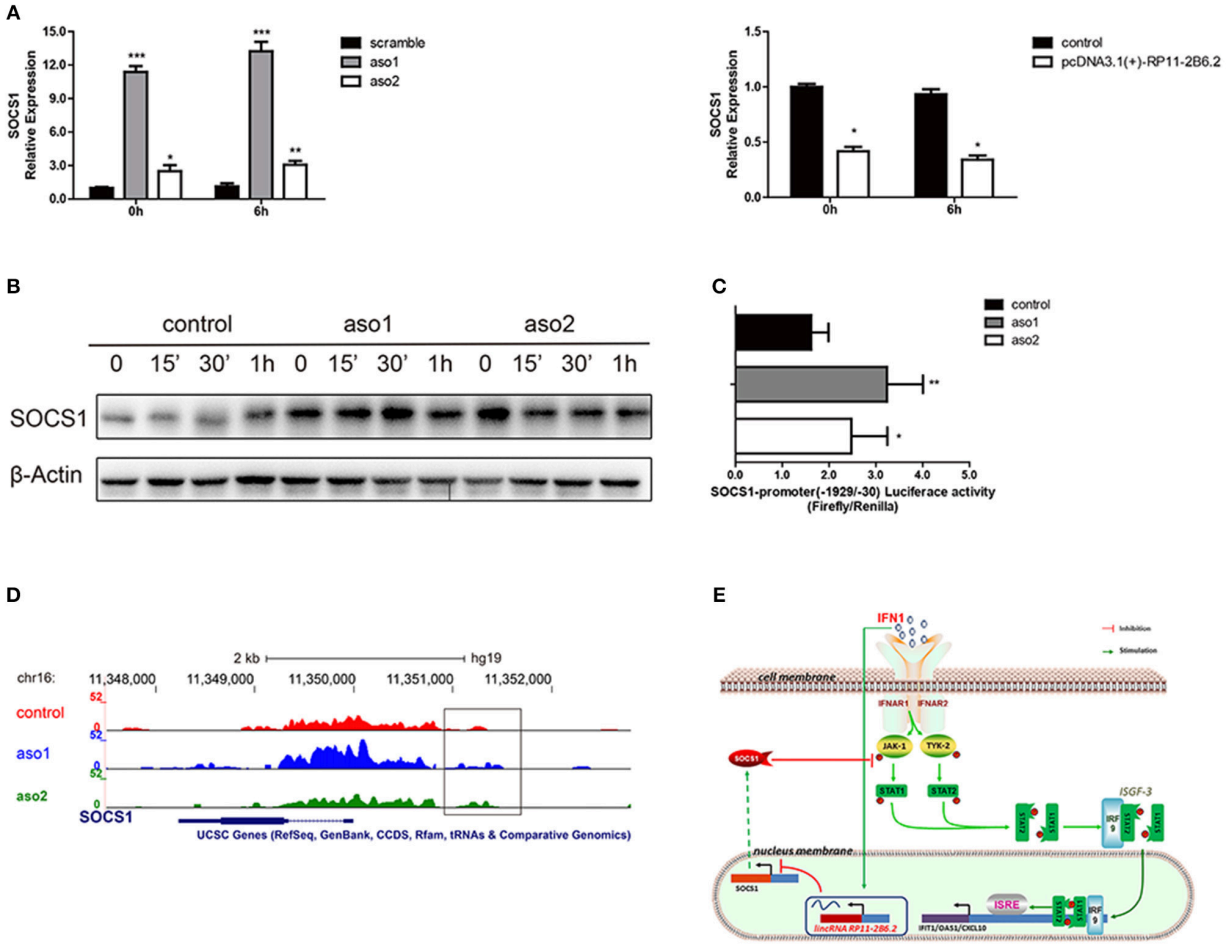
LncRNA RP11-2B6.2 Suppresses the Transcription of Negative Regulator SOCS1 in IFN-I Pathway. **(A)**The mRNA level of SOCS1 in HeLa cells with lncRNA RP11-2B6.2 ASOs or scramble, lncRNA RP11-2B6.2 overexpression or empty pcDNA 3.1(+)-5′-HA vector, were determined by qPCR. **(B)** The protein level of SOCS1 in HeLa cells with lncRNA RP11-2B6.2 ASOs or scramble, were determined by western blotting. **(C)** The promoter activity of SOCS1 in HeLa cells with lncRNA RP11-2B6.2 ASOs or scramble, were determined by dual-luciferase reporter assay. **(D)** The chromatin accessibility of SOCS1 in HeLa cells with lncRNA RP11-2B6.2 ASOs or scramble, were determined by normalized ATAC sequencing analysis. **(E)** Schematic diagram of the mechanism by which lncRNA RP11-2B6.2 positively regulates the IFN-activated classical JAK-STAT signaling pathway. IFN-I triggered an unknown transcriptional factor to induce lncRNA RP11-2B6.2 expression. LncRNA RP11-2B6.2 subsequently enhanced the IFN-I-induced phosphorylation of TYK2, JAK1, and STAT1 through attenuating of SOCS1 at the transcriptional level, and eventually leading to the increased expression of IFN-I stimulated inflammatory genes. ^*^P < 0.05, ^**^P < 0.01, ^***^P < 0.001.

To study the mechanism for the regulatory function of lncRNA RP11-2B6.2 in IFN-I signaling pathway, we first assessed the protein-coding potential of lncRNA RP11-2B6.2. We have used two on-line tools developed by different labs (28, 29) and the results showed no protein-coding potential for lncRNA RP11-2B6.2 (data not shown). Our findings indicate that lncRNA RP11-2B6.2 is a positive regulator of IFN-I signaling pathway. To identify the molecular mechanisms for lncRNA RP11-2B6.2's function, we tested if lncRNA RP11-2B6.2 could affect the expression of currently known negative regulators of IFN-I signaling pathway, such as SOCS1, SHP1, SHP2, and PTP1B (http://lsresearch.thomsonreuters.com/maps/429/) (30, 31). We found only mRNA levels of SOCS1 showed a modest change after lncRNA RP11-2B6.2 interruption (Figure 4A and Supplementary Figure 8). Consistently, lncRNA RP11-2B6.2 deficiencies enhanced the expression of SOCS1 protein (Figure 4B).

As lncRNA RP11-2B6.2 showed a punctate aggregation distribution in the nucleus of HeLa cells, we supposed that it might function to regulate the transcription of SOCS1 gene (Supplementary Figure 9). To testify this hypothesis, we investigated whether lncRNA RP11-2B6.2 affected the transcriptional activity of SOCS1 promoter through luciferase reporter assay. The results showed that the downregulation of lncRNA RP11-2B6.2 caused a considerable increase of SOCS1 promoter activity (Figure 4C). We further evaluated the chromatin accessibility status upstream of SOCS1 gene by ATAC sequencing. We found that the downregulation of lncRNA RP11-2B6.2 favored an open chromatin status upstream of SOCS1 gene (Figure 4D).

These findings indicate that lncRNA RP11-2B6.2 may epigenetically affects the transcription of SOCS1 to positively regulate IFN-I signaling pathway (Figure 4E).

## Discussion

So far, lncRNA profiles have been done mainly in immune cells of SLE patients (32, 33). However, considering SLE-related organ damage, lncRNAs in tissue resident cells may also contribute to the abnormal activation of inflammatory signaling pathways. To explore the role of lncRNAs in kidney tissues of LN, we analyzed the expression profiles of lncRNAs in kidney biopsies of LN patients by RNA sequencing. We found that there was a differential expression of lncRNAs between LN kidney tissue samples and controls, which indicates lncRNAs in renal resident cells may participate in the disorders of LN.

Next, we selected lncRNA RP11-2B6.2, which increased in the kidney biopsies of LN patients, for further investigation by two criteria: (1) lncRNAs which had the most significant *P*-value (top 5 lowest); (2) lncRNAs which had an association of its expression levels with those of ISGs. Renal resident cells have been demonstrated to contribute to the local imbalance of pro-/anti-inflammatory cytokine responses induced by the deposition of immune complexes (ICs) in the kidney of LN (3, 34, 35). Among these cells, both renal mesangial cells and tubular epithelial cells contribute to disease pathogenesis, such as mesangial proliferation and interstitial fibrosis causing kidney impairment (3, 36). We found lncRNA RP11-2B6.2 was expressed both in glomerulus and tubules by RNA scope method. Additionally, we measured the expression of lncRNA RP11-2B6.2 in PBMCs of LN patients. Although, lncRNA RP11-2B6.2 was also expressed and upregulated in PBMCs of LN patients compared to healthy controls, the difference was not that striking as seen in kidney tissues. Thus, the above data suggest lncRNA RP11-2B6.2 may be important in kidney tissues in LN. Subsequently, we found that lncRNA RP11-2B6.2 could be induced by IFN-I in HRMCs and HK2 cells, which suggests that the over-activation of IFN-I signaling pathway may be one of the reasons for the upregulation of lncRNA RP11-2B6.2 in LN kidney tissues.

Finally, we tested if lncRNA RP11-2B6.2 was involved in regulating IFN-I signaling pathway. Alteration of the expression levels of lncRNA RP11-2B6.2, as shown in gain or loss of function experiments, affected the induction of ISGs and the phosphorylation of signaling components downstream of IFN-I signaling pathway in renal cells. Cells spontaneously use various mechanisms to fine-tune the IFN-I signaling pathway to avoid tissue damages and autoimmune diseases. To explore the mechanism for lncRNA RP11-2B6.2 in regulating IFN-I signaling, we tested if lncRNA RP11-2B6.2 affected the expression of currently known negative regulators of IFN-I signaling pathway, such as SOCS1, SHP1, SHP2, and PTP1B. We showed that lncRNA RP11-2B6.2 could inhibit the expression of SOCS1. Additionally, we showed that lncRNA RP11-2B6.2 inhibited SOCS1 expression by reducing the chromatin accessibility upstream of SOCS1 gene and by inhibiting the promoter activity of SOCS1 gene. SOCS1, also called SSI-1 (STAT-induced STAT inhibitor-1) and JAB-1 (JAK binding protein-1), is a pivotal member in the suppressor of cytokine signaling family, which can restrain the activity of IFNAR1 and the phosphorylation of TYK2, JAK1, STAT1 (28, 37, 38). Moreover, it has been reported that SOCS1 is downregulated in glomerular mesangial cells and tubular epithelial cells in lupus mice (39), and inadequate induction of SOCS1 causes kidney damage via enhanced IFN responses in mice (40). Our results indicate that SOCS1 might be a target molecule of lncRNA RP11-2B6.2 for its function as a positive regulator of IFN-I signaling pathway.

Our study has limitations. Although we have pointed out that lncRNA RP11-2B6.2 could regulate the expression of SOCS1 epigenetically, we still don't know the exact mechanism. Since we have ruled out the possibility for lncRNA RP11-2B6.2 to produce functional proteins, we will further explore its function as an RNA mediator of epigenetic changes. Another limitation is that although we have showed that lncRNA RP11-2B6.2 was expressed in renal glomerular cells and tubular cells and the differential expression of lncRNA RP11-2B6.2 was much smaller in PBMCs than that seen in kidney biopsies, we cannot make a conclusion that lncRNA RP11-2B6.2 functions only in kidney resident cells in LN. However, based on our data, we propose that lncRNA RP11-2B6.2 mainly functions in kidney resident cells in LN mediating the overactivation of IFN-I signaling pathway.

In summary, our data demonstrate that there is a dysregulated expression of lncRNAs in kidney tissues of LN; lncRNA RP11-2B6.2 is increased in renal tissues of LN patients and contributes to the over-activation of IFN-I signaling pathway in renal cells by epigenetically inhibiting the expression of SOCS1. These findings shed a new light on the expression and function of lncRNAs in kidney tissues of LN, while lncRNA RP11-2B6.2 may be used as a new therapeutic target for the intervention of over-activated IFN-I responses.

## Author Contributions

All authors were involved in drafting the article or revising it critically for important intellectual content, and all authors approved the final version to be published. ZL, BQ, and NS conceived and designed the experiments, ZL, ZY, ZX, LW, and YO performed the experiments, ZL, ZX, CY, CC, NX, and JM analyzed and interpreted the data, GH, JW, YM, ZY, YL, JQ, CZ, HD, and QG contributed reagents, materials,and analysis tools.

### Conflict of Interest Statement

The authors declare that the research was conducted in the absence of any commercial or financial relationships that could be construed as a potential conflict of interest.

## References

[1] Lisnevskaia L Murphy G Isenberg D. Systemic lupus erythematosus. Lancet. (2014) 384:1878–88. 10.1016/S0140-6736(14)60128-8 24881804

[2] Tsokos GC. Systemic lupus erythematosus. N Engl J Med. (2011) 365:2110–21. 10.1056/NEJMra1100359 22129255

[3] de Zubiria Salgado A Herrera-Diaz C Lupus nephritis: an overview of recent findings. Autoimmune Dis. (2012) 2012:849684 10.1155/2012/849684 22536486 PMC3318208

[4] Hahn BH McMahon MA Wilkinson A Wallace WD Daikh DI Fitzgerald JD. American College of Rheumatology guidelines for screening, treatment, and management of lupus nephritis. Arthritis Care Res. (2012) 64:797–808. 10.1002/acr.21664 22556106 PMC3437757

[5] Somers EC Richardson BC. Environmental exposures, epigenetic changes and the risk of lupus. Lupus. (2014) 23:568–76. 10.1177/0961203313499419 24763540 PMC4000546

[6] Liu Z Davidson A. Taming lupus—a new understanding of pathogenesis is leading to clinical advances. Nat Med, (2012) 18:871–82. 10.1038/nm.2752 22674006 PMC3607103

[7] Quinn JJ Chang HY. Unique features of long non-coding RNA biogenesis and function. Nat Rev Genetics. (2016) 17:47–62. 10.1038/nrg.2015.10 26666209

[8] Ponting CP Oliver PL Reik W. Evolution and functions of long noncoding RNAs. Cell. (2009) 136:629–41. 10.1016/j.cell.2009.02.006 19239885

[9] Wu Y Zhang F Ma J Zhang X Wu L Qu B. Association of large intergenic noncoding RNA expression with disease activity and organ damage in systemic lupus erythematosus. Arthr Res Ther. (2015) 17:131. 10.1186/s13075-015-0632-3 25994030 PMC4440330

[10] Zhang F Wu L Qian J Qu B Xia S La T. Identification of the long noncoding RNA NEAT1 as a novel inflammatory regulator acting through MAPK pathway in human lupus. J Autoimmunity. (2016) 75:96–104. 10.1016/j.jaut.2016.07.012 27481557

[11] Ronnblom L Eloranta ML Alm GV. The type I interferon system in systemic lupus erythematosus. Arthritis Rheumat. (2006) 54:408–20. 10.1002/art.21571 16447217

[12] Banchereau J Pascual V Type I interferon in systemic lupus erythematosus and other autoimmune diseases. Immunity. (2006) 25:383–92. 10.1016/j.immuni.2006.08.010 16979570

[13] Nacionales DC Kelly-Scumpia KM Lee PY Weinstein JS Lyons R Sobel E. Deficiency of the type I interferon receptor protects mice from experimental lupus. Arthr Rheumat. (2007) 56:3770–83. 10.1002/art.23023 17968932 PMC2909118

[14] Santiago-Raber ML Baccala R Haraldsson KM Choubey D Stewart TA Kono DH. Type-I interferon receptor deficiency reduces lupus-like disease in NZB mice. J Exp Med. (2003) 197:777–88. 10.1084/jem.20021996 12642605 PMC2193854

[15] Ikeda K Hayakawa K Fujishiro M Kawasaki M Hirai T Tsushima H. JAK inhibitor has the amelioration effect in lupus-prone mice: the involvement of IFN signature gene downregulation. BMC Immunol. (2017) 18:41. 10.1186/s12865-017-0225-9 28830352 PMC5568047

[16] Zheng B Yu XQ Greth W Robbie GJ Population pharmacokinetic analysis of sifalimumab from a clinical phase IIb trial in systemic lupus erythematosus patients. Br J Clin Pharmacol. (2016) 81:918–28. 10.1111/bcp.12864 26659791 PMC4834601

[17] Furie R Khamashta M Merrill JT Werth VP Kalunian K Brohawn P. Anifrolumab, an Anti-Interferon-alpha receptor monoclonal antibody, in moderate-to-severe systemic lupus erythematosus. Arthr Rheumatol. (2017) 69:376–86. 10.1002/art.39962 28130918 PMC5299497

[18] Petri M Orbai AM Alarcon GS Gordon C Merrill JT Fortin PR. Derivation and validation of the systemic lupus international collaborating clinics classification criteria for systemic lupus erythematosus. Arthr Rheumat. (2012) 64:2677–86. 10.1002/art.34473 22553077 PMC3409311

[19] Gladman DD Ibanez D Urowitz MB. Systemic lupus erythematosus disease activity index 2000. J Rheumatol. (2002) 29:288–91. 10.1097/00124743-200202000-00018 11838846

[20] Bajema IM Wilhelmus S Alpers CE Bruijn JA Colvin RB Cook HT. Revision of the International Society of Nephrology/Renal Pathology Society classification for lupus nephritis: clarification of definitions, and modified National Institutes of Health activity and chronicity indices. Kidney Int. (2018) 93:789–96. 10.1016/j.kint.2017.11.023 29459092

[21] Gilbert LA Horlbeck MA Adamson B Villalta JE Chen Y Whitehead EH. Genome-scale crispr-mediated control of gene repression and activation. Cell. (2014) 159:647–61. 10.1016/j.cell.2014.09.029 25307932 PMC4253859

[22] George TC Fanning SL Fitzgerald-Bocarsly P Medeiros RB Highfill S Shimizu Y. Quantitative measurement of nuclear translocation events using similarity analysis of multispectral cellular images obtained in flow. J Immunol Methods. (2006) 311:117–29. 10.1016/j.jim.2006.01.018 16563425

[23] Buenrostro JD Wu B Chang HY Greenleaf WJ. ATAC-seq: a method for assaying chromatin accessibility genome-wide. Curr Protoc Mol Biol. (2015) 109:21.29.1–9. 10.1002/0471142727.mb2129s109 25559105 PMC4374986

[24] Feng X Wu H Grossman JM Hanvivadhanakul P FitzGerald JD Park GS. Association of increased interferon-inducible gene expression with disease activity and lupus nephritis in patients with systemic lupus erythematosus. Arthr Rheumat. (2006) 54:2951–62. 10.1002/art.22044 16947629

[25] Yao Y Higgs BW Morehouse C de los Reyes M Trigona W Brohawn P. Development of potential pharmacodynamic and diagnostic markers for anti-IFN-α monoclonal antibody trials in systemic lupus erythematosus. Hum Genom Proteom. (2009) 2009:374312. 10.4061/2009/374312 20948567 PMC2950308

[26] Baechler EC Batliwalla FM Karypis G Gaffney PM Ortmann WA Espe KJ. Interferon-inducible gene expression signature in peripheral blood cells of patients with severe lupus. Proc Natl Acad Sci U.S.A. (2003) 100:2610–5. 10.1073/pnas.0337679100 12604793 PMC151388

[27] Platanias LC. Mechanisms of type-I- and type-II-interferon-mediated signalling. Nat Rev Immunol. (2005) 5:375–86. 10.1038/nri1604 15864272

[28] Wang L Park HJ Dasari S Wang S Kocher JP Li W. CPAT: Coding-potential assessment tool using an alignment-free logistic regression model. Nucleic Acids Res. (2013) 41:e74. 10.1093/nar/gkt006 23335781 PMC3616698

[29] Kong L Zhang Y Ye ZQ Liu XQ Zhao SQ Wei L. CPC: assess the protein-coding potential of transcripts using sequence features and support vector machine. Nucleic Acids Res. (2007) 35(Web Server issue): W345–9. 10.1093/nar/gkm391 17631615 PMC1933232

[30] Fenner JE Starr R Cornish AL Zhang JG Metcalf D Schreiber RD. Suppressor of cytokine signaling 1 regulates the immune response to infection by a unique inhibition of type I interferon activity. Nat Immunol. (2006) 7:33–9. 10.1038/ni1287 16311601

[31] Witte S Muljo SA. Integrating non-coding RNAs in JAK-STAT regulatory networks. Jak Stat. (2014) 3:e28055. 10.4161/jkst.28055 24778925 PMC3995732

[32] Li LJ Zhao W Tao SS Li J Xu SZ Wang JB. Comprehensive long non-coding RNA expression profiling reveals their potential roles in systemic lupus erythematosus. Cell Immunol. (2017) 319:17–27. 10.1016/j.cellimm.2017.06.004 28622785

[33] Luo Q Li X Xu C Zeng L Ye J Guo Y. Integrative analysis of long non-coding RNAs and messenger RNA expression profiles in systemic lupus erythematosus. Mol Med Rep. (2018) 17:3489–96. 10.3892/mmr.2017.8344 29286106 PMC5802165

[34] Bagavant H Fu SM. Pathogenesis of kidney disease in systemic lupus erythematosus. Curr Opin Rheumatol. (2009) 21:489–94. 10.1097/BOR.0b013e32832efff1 19584729 PMC2841319

[35] Davidson A Aranow C. Lupus nephritis: lessons from murine models. Nat Rev Rheumatol. (2010) 6:13–20. 10.1038/nrrheum.2009 19949431 PMC4120882

[36] Yung S Cheung KF Zhang Q Chan TM. Mediators of inflammation and their effect on resident renal cells: implications in lupus nephritis. Clin Dev Immunol. (2013) 2013: 317682. 10.1155/2013/317682 24171032 PMC3793320

[37] Wang H Wang J Xia Y. Defective suppressor of cytokine signaling 1 signaling contributes to the pathogenesis of systemic lupus erythematosus. Front Immunol. (2017) 8:1292. 10.3389/fimmu.2017.01292 29085365 PMC5650678

[38] Nakagawa R Naka T Tsutsui H Fujimoto M Kimura A Abe T. SOCS-1 participates in negative regulation of LPS responses. Immunity. (2002) 17:677–87. 10.1016/S1074-7613(02)00449-1 12433373

[39] Dong J Wang QX Zhou CY Ma XF Zhang YC. Activation of the STAT1 signalling pathway in lupus nephritis in MRL/lpr mice. Lupus. (2007) 16:101–9. 10.1177/096 1203306075383 17402366

[40] Fujimoto M Tsutsui H Xinshou O Tokumoto M Watanabe D Shima Y. Inadequate induction of suppressor of cytokine signaling-1 causes systemic autoimmune diseases. Int Immunol. (2004) 16:303–14. 10.1093/intimm/dxh030 14734616

