# Supplementary material for: Identification of Renal Long Non-coding RNA RP11-2B6.2 as a Positive Regulator of Type I Interferon Signaling Pathway in Lupus Nephritis
Source: Front Immunol. 2019 May 3;10:975. doi: 10.3389/fimmu.2019.00975 (PMC6509587; doi:10.3389/fimmu.2019.00975)
Supplement: Supplementary file 1 [file Data_Sheet_1.docx]

Supplementary Material

# Supplementary Tables

**Supplementary Table 1. Sequences of qPCR Primers** **Used in the Study**

| Gene name | forward (5’-3’) | reverse (5’-3’) |
| --- | --- | --- |
| GAPDH | CTCCTCCTGTTCGACAGTCA | CAATACGACCAAATCCGTTG |
| RP11-2B6.2 | AGGTTGATTGAGCCAGACTTT | GTTTGCCCATCGGAGGA |
| IFIT1 | TTGCTGAAGTGTGGAGGAAA | CCAGGCGATAGGCAGAGAT |
| OAS1 | CACAGCCTCACTTCATTCCA | GACATTACCCTCCCATCAGG |
| CXCL10 | TTCTGATTTGCTGCCTTATC | CTTGGATTAACAGGTTGATTACT |
| SOCS1 | GCCCCTTCTGTAGGATGGT | CTGCTGTGGAGACTGCATTG |
| PTP1B | ATACCACATGGCCTGACTTT | CCAGACAGAAGGTTCCAGAC |
| SHP1 | GACTTCGTGCTTTCTGTGC | TGGCGATGTAGGTCTTAGC |
| SHP2 | GGGAAAGAAGCAGAGAAATT | CACTTTAGACTTGCCGTCAT |

**Supplementary Table 2. Antibodies of Western Blotting Used in the Study**

| Antibody | Dilution | Source |
| --- | --- | --- |
| rabbit anti-phosphorylated-STAT1 antibody (Tyr701) | 1:1000 | Cell Signaling Technology |
| mouse anti-STAT1 antibody | 1:200 | Santa Cruz |
| rabbit anti-phosphorylated-JAK1 antibody (Tyr1022/1023) | 1:1000 | Cell Signaling Technology |
| rabbit anti-JAK1 antibody | 1:200 | Santa Cruz |
| rabbit anti-phosphorylated-TYK2 antibody (Tyr1054/1055) | 1:1000 | Cell Signaling Technology |
| rabbit anti-TYK2 antibody | 1:1000 | Cell Signaling Technology |
| rabbit anti-SOCS1 antibody | 1:1000 | Abcam |
| mouse anti-β-actin antibody | 1:10000 | Cell Signaling Technology |
| anti-mouse-HAP-linked secondary antibody | 1:5000 | Cell Signaling Technology |
| anti-rabbit-HAP-linked secondary antibody | 1:5000 | Cell Signaling Technology |

**Supplementary Table 3.** **Differentially Expressed LncRNAs with Top 5 Significance in LN Patients.**

| LncRNA | patients vs controls | | correlation to IFN score | |
| --- | --- | --- | --- | --- |
|  | fold change | *P*-value | Spearman R | *P*-value |
| TCONS_00002573 | 3.300441 | 1.32E-10 | -0.082 | 0.717 |
| TCONS_00023603 | 4.806713 | 2.99E-09 | -0.081 | 0.721 |
| TCONS_00012375 | 4.619398 | 8.78E-09 | -0.060 | 0.789 |
| ***RP11-2B6.2*** | ***5.463333*** | ***5.76E-08*** | ***0.430**** | ***0.046*** |
| TCONS_00031374 | 3.044656 | 5.65E-07 | -0.202 | 0.368 |

# Supplementary Figures

**
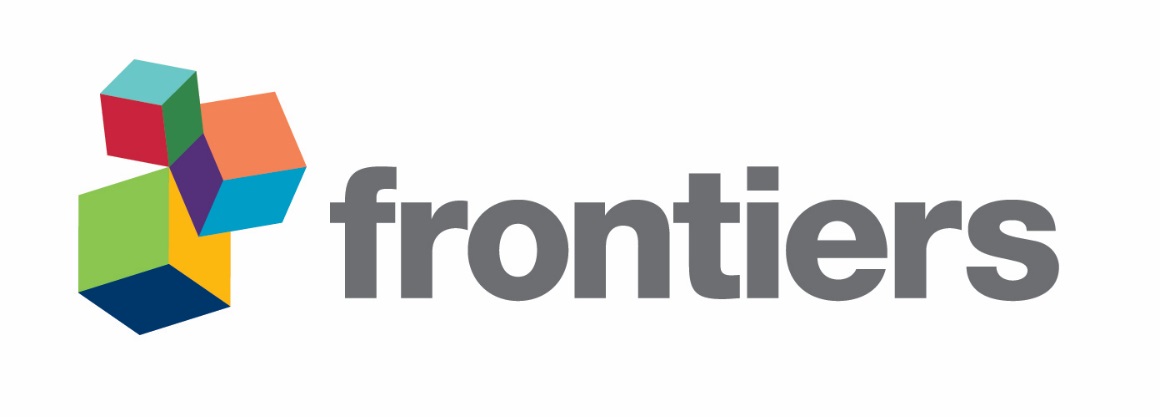
**

**Supplementary Figure 1.** **Increased Expression of IFN-I Stimulated Genes in LN Patients.**


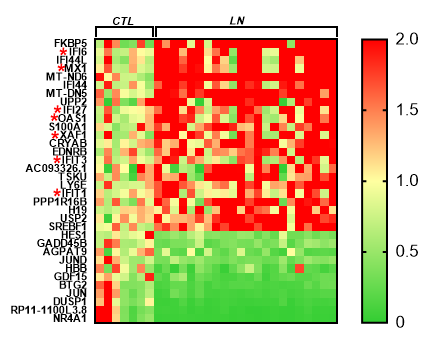


23 upregulated mRNAs and 11 downregulated mRNAs showed differential expression in transcriptome profiling of renal tissues from 22 LN patients compared to 7 controls (≥2-fold change and *P*<0.05). *IFN-I stimulated genes.

**Supplementary Figure 2. Independent Expression of LncRNA RP11-2B6.2 from Medication Use.**


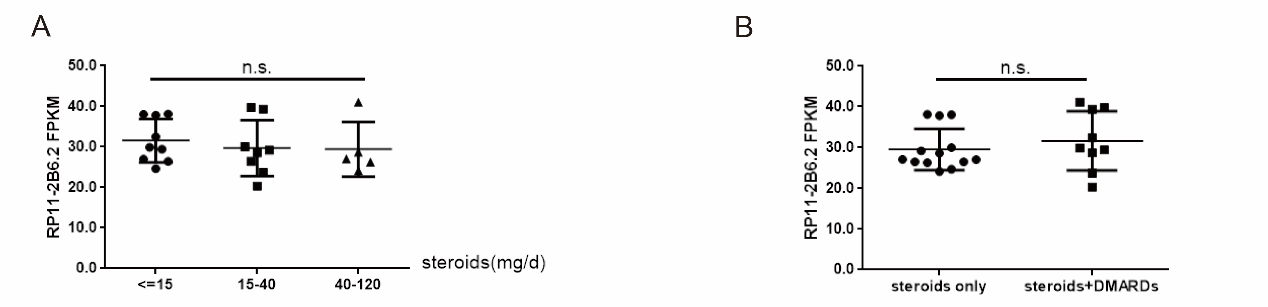


Patients were classified based on use of steroids (A) and disease modifying anti-rheumatic drugs (DMARDs) (B), and the expression of lincRNA RP11-2B6.2 in LN patients with different medications was presented. Horizontal bars indicated the mean value. n.s. no significant difference.

**Supplementary Figure 3. Increased Expression Levels of LncRNA RP11-2B6.2 in PBMCs and Kidney Tissues of Lupus Patients.**


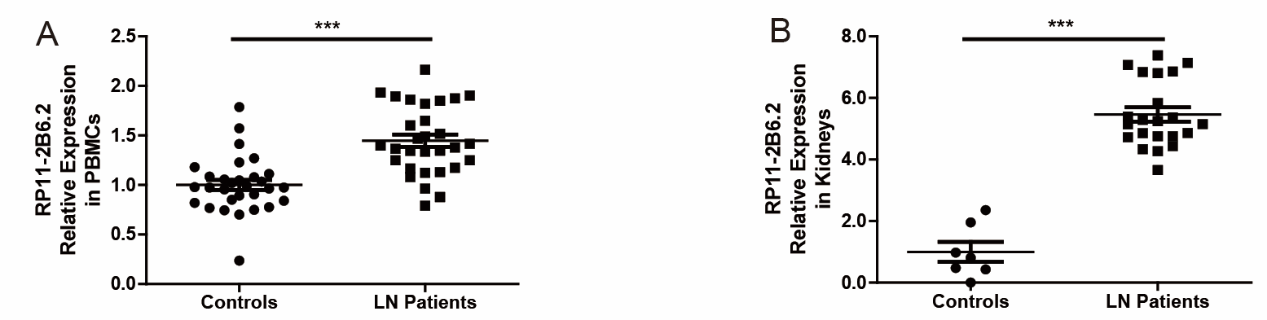


The expression of lncRNA RP11-2B6.2 in PBMCs (A) of LN patients and healthy controls was measured by qPCR. The expression of lncRNA RP11-2B6.2 in kidney tissues (B) of LN patients and healthy controls is plotted using RNA sequencing data. Relative expression of lncRNA RP11-2B6.2 is presented. Horizontal bars indicate the means of each group. ****P*<0.001.

**Supplementary Figure 4. Elevated LncRNA RP11-2B6.2 Levels by IFN-I Stimulation in HeLa Cells and Renal Cells.**

Induction of lincRNA RP11-2B6.2 by IFN-I (1000 U/ml) in HeLa cells (A), HRMCs (B), and HK2 cells (C) measured at 0, 0.5, 1, 3, 6, and 12 h post-stimulation respectively. Relative mRNA levels based on qPCR results were calculated using the endogenous GAPDH levels. Values were reported as 2^-ΔΔCT^ fold change. **P*<0.05, ***P*<0.01.


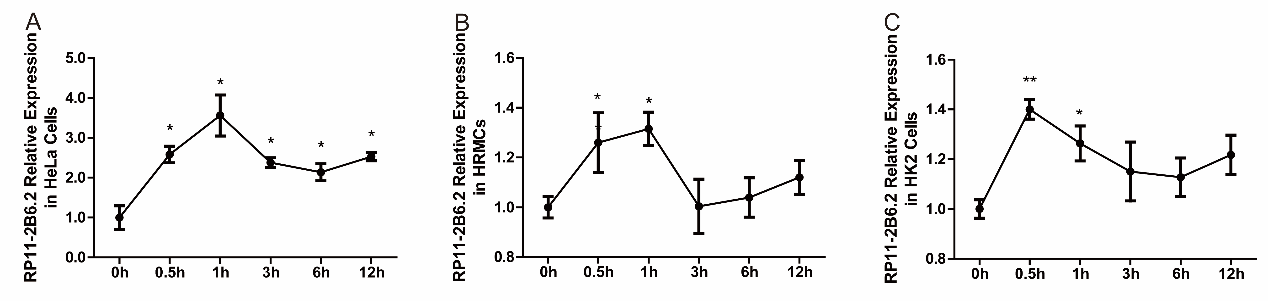


**Supplementary Figure 5. Spatial Distribution of LncRNA RP11-2B6.2 in Renal Tissues of LN Patients.**

**
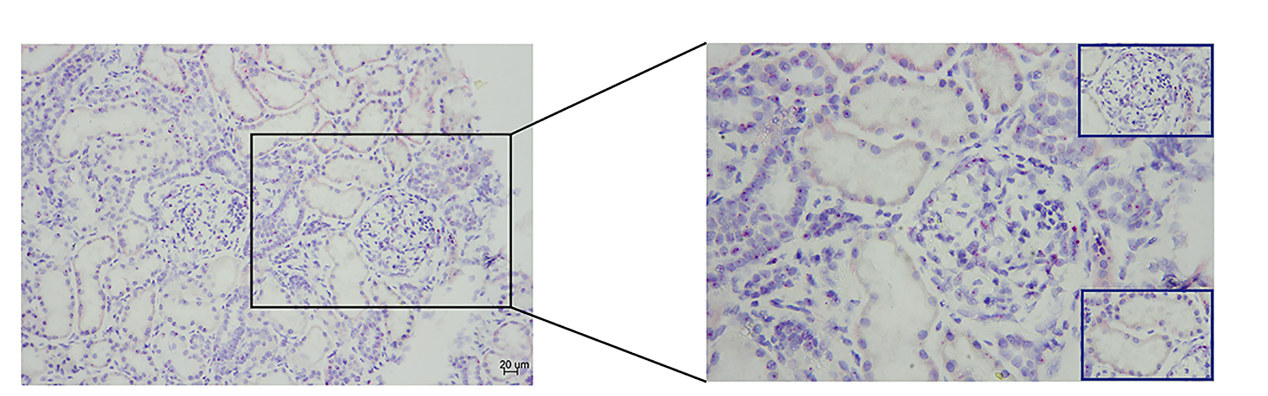
**

Spatial distribution of lncRNA RP11-2B6.2 in renal tissues of LN patients using RNAscope. Left: magnification ×200; right: magnification ×400; upper right: glomerulus; lower right: tubules.

**Supplementary Figure 6. Overview of the Landscape across LncRNA RP11-2B6.2 Modulatory Genes**


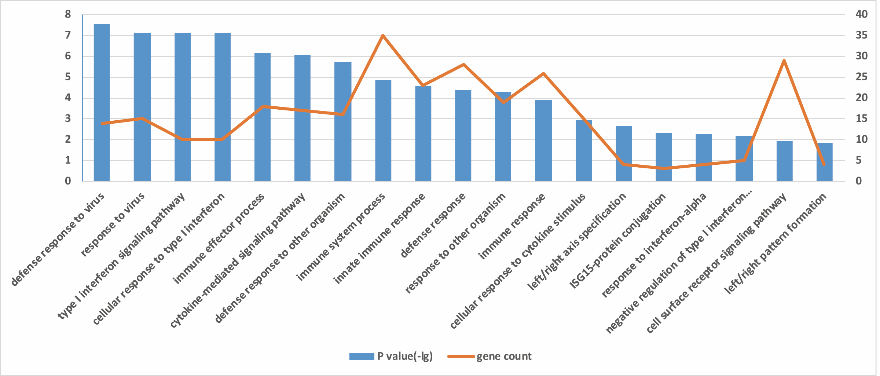


GO enrichment analysis of lincRNA RP11-2B6.2-regulated genes from RNA sequencing result in HeLa cells. Figures were constructed using relevant gene counts and *P*-values of various functional factors.

**Supplementary Figure 7. Sketches of Primers, ASOs and sgRNAs for LncRNA RP11-2B6.2.**


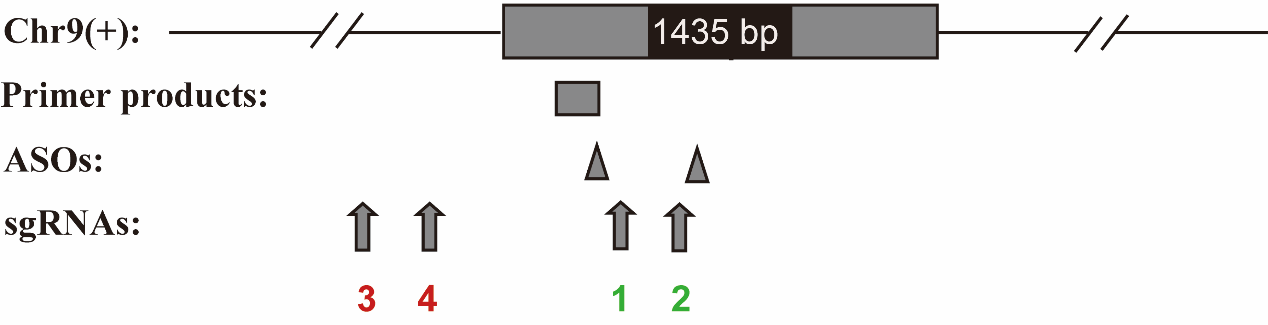


Graphical representation of full-length human lincRNA RP11-2B6.2 transcript (1435 bp) was shown, qPCR primers, ASOs and sgRNAs targeting lincRNA RP11-2B6.2 were indicated.

**Supplementary Figure 8.** **Regulation of Multiple Key Regulators in IFN-I Pathway by LncRNA RP11-2B6.2.**


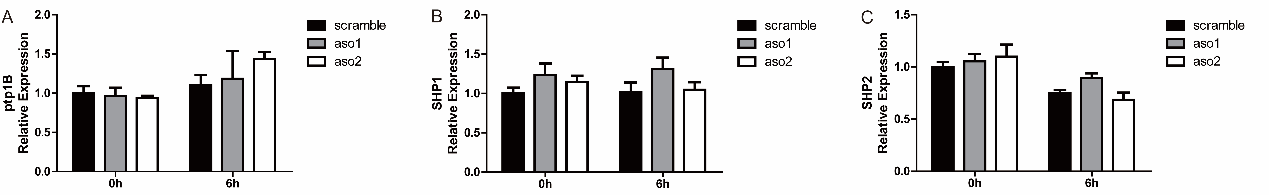


The PTP1B (A), SHP1 (B), and SHP2 (C) mRNA expression in IFN-I-stimulated HeLa cells transfected with lincRNA RP11-2B6.2 ASOs or scramble, were determined by qPCR. Values were means ± SEM from three independent experiments.

**Supplementary Figure 9. Nucleus and Plasma Distribution of LncRNA RP11-2B6.2 in HeLa Cells.**

**
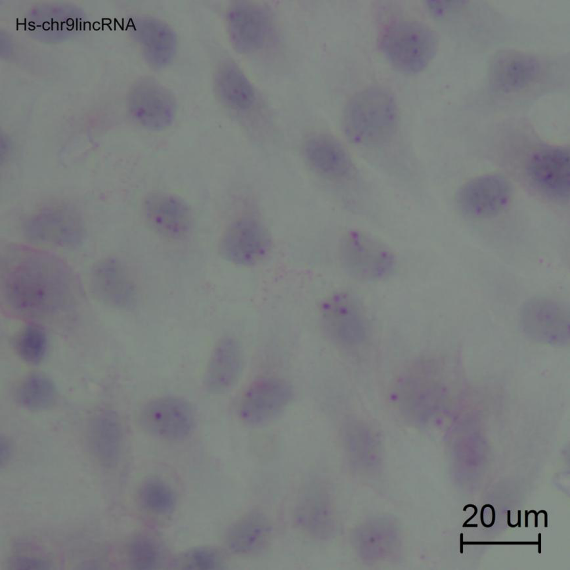
**

Nucleus and plasma distribution of lincRNA RP11-2B6.2 in HeLa cells were measured by RNAscope and HE staining. Scale bar 20 μm.
